# Elastic Compression Attenuates Inflammatory Response in a Model of Acute Subcutaneous Abdominal Inflammation

**DOI:** 10.1007/s00266-026-05983-0

**Published:** 2026-06-22

**Authors:** Rubya Pereira Zaccaron, Karina Borges Ferreira, Ligia Milanez Venturini, Laura de Roch Casagrande, Camila da Costa, Gabrielli Martins, Igor Ramos Lima, Carolini Mendes, Rahisa Scussel, Ricardo Andrez Machado-De-Ávila, Anand Thirupathi, Paulo Cesar Lock Silveira

**Affiliations:** 1 Laboratory of Experimental Physiopathology, Program of postgraduate in Science of Health, Universidade do Extremo Sul Catarinense, Criciúma, Santa Catarina 88806-000 Brazil; 2 Faculty of Sports Science, Ningbo University, Ningbo, 315211 China

**Keywords:** Plastic surgery, Elastic bandage, Inflammation, Mechanomodulation

## Abstract

**Background:**

Acute inflammation, when unresolved, can lead to complications that impair tissue repair and therapeutic outcomes.

**Objective:**

In this study, we employed a model of lipopolysaccharide (LPS)-induced acute subcutaneous abdominal inflammation in mice to investigate the modulatory effects of elastic compression.

**Methods:**

LPS administration elicited a robust inflammatory response, characterized by increased leukocyte infiltration, edema, and upregulation of pro-inflammatory mediators. Elastic compression significantly attenuated this response, reducing leukocyte counts in subcutaneous lavage, histological inflammatory infiltrates, and the expression of key pro-inflammatory genes and proteins, including NF-κB, IL-1β, and TNF-α, at both 24 and 72 hours post-induction. Mechanistically, these effects may result from the compressive force altering microvascular dynamics and modulating macrophage polarization and mechanotransduction pathways, including TLR4 and integrin signaling. Additionally, compression preserved redox homeostasis, as indicated by stable oxidative stress markers and antioxidant responses. To our knowledge, this is the first study to demonstrate that elastic compression modulates inflammation at molecular, cellular, and tissue levels in an acute inflammation model.

**Results:**

These findings support the therapeutic potential of elastic compression as a non-pharmacological strategy for managing acute inflammation, with possible applications in postoperative care, traumatic edema, and other soft tissue inflammatory conditions.

**Conclusion:**

Further translational and clinical studies are warranted to validate these outcomes and guide evidence-based application protocols.

**No Level Assigned:**

This journal requires that authors assign a level of evidence to each submission to which Evidence-Based Medicine rankings are applicable. This excludes Review Articles, Book Reviews, and manuscripts that concern Basic Science, Animal Studies, Cadaver Studies, and Experimental Studies. For a full description of these Evidence-Based Medicine ratings, please refer to the Table of Contents or the online Instructions to Authors www.springer.com/00266.

## Introduction

Recent advances in techniques and biomaterials have led to the popularization of both plastic and reconstructive surgical interventions [1]. However, the scientific community still debated the impact of these surgeries on tissues, especially concerning the trauma caused by the interventions and the ensuing inflammatory response [2–4].

Subcutaneous tissue manipulation, although carefully controlled, induces a natural inflammatory response, which is essential for the healing process. A series of complex events initiates the healing process, such as hemostasis, cell proliferation, and tissue remodeling, all of which are essential for recovery. Nonetheless, when exacerbated or prolonged, the inflammatory process can result in complications, such as hypertrophic scar formation, fibrosis, and fluid accumulation in the form of seroma [5, 6]. These effects can significantly impact patient satisfaction and the success of esthetic results, which leads to an increasing interest in strategies to mitigate and optimize recovery processes [7, 8].

Physiotherapy has explored several approaches to modulate clinical responses to inflammation in order to improve recovery processes, including the use of elastic compression bandages in the postoperative period. Elastic compression bandages are widely used in clinical practice because they provide compression and support, which contribute to reducing edema and pain [9]. Although clinical outcomes demonstrate clear benefits, the biochemical mechanisms underlying these effects remain incompletely elucidated. The compression exerted by the bandage may stimulate mechanoreceptors and modulate the inflammatory response [10, 11]; however, the precise interactions between these mechanical stimuli and the cellular processes involved in tissue repair have not been thoroughly investigated [12, 13].

Given this context, it is essential to investigate the biochemical mechanisms activated by elastic compression bandaging, as compression therapy, and then identify the molecular pathways involved in modulating the inflammatory process and potentially accelerating tissue repair. This may also contribute to optimizing and promoting its application in clinical practice. Therefore, this study aims to evaluate the effects of elastic compression bandaging on biochemical, molecular, and histological markers of inflammation.

## Materials and Methods

### Ethical Approvals

The Ethics Committee on the Use of Animals of UNESC (Comissão de Ética no Uso de Animais—CEUA/UNESC) approved this study under the protocol number 46/2022. All procedures were conducted in accordance with Brazilian legislation on the use of animals for scientific and educational purposes (Law 11.794, DOU 27/05/2013, MCTI, p. 7), and complied with the guidelines of the National Council for the Control of Animal Experimentation (Conselho Nacional de Controle de Experimentação Animal—CONCEA) and the National Commission for Animal Welfare (Comissão Nacional de Bem-Estar Animal—COBEA). Additionally, all procedures adhered to the ARRIVE (Animal Research: Reporting of In Vivo Experiments) guidelines.

### Animals and Experimental Design

A total of 96 Wistar rats were used and divided into two main groups of 48 animals each, corresponding to euthanasia at 24 hours and 72 hours post-intervention. Each main group was further subdivided into four experimental groups (n = 12): Sham, LPS, Elastic Compression (EC), and LPS + EC. Following anesthesia and abdominal area preparation, a single subcutaneous injection of lipopolysaccharide (LPS; 15 mg/kg) was administered into the lower right quadrant of the abdomen in the designated groups to induce inflammation, while the Sham group received an equivalent volume of saline solution (0.9%) [14].

In the groups assigned to receive elastic compression, Theraband Kinesiology Tape® was applied with 50% stretch. Two strips measuring 5 cm × 12 cm were positioned from the abdominal to the dorsal region to cover the area of interest.

At the designated time points (24h and 72h), the subcutaneous tissue was washed for cell counting. Immediately following euthanasia, the subcutaneous tissue from the lower right quadrant was carefully excised, excluding the superficial skin, fascia, and underlying muscle tissue. The collected samples were then further submitted for histological, molecular, and biochemical analyses.

### Leukocyte Counting in Subcutaneous Lavage

To assess the migration of inflammatory cells, subcutaneous lavage was performed using phosphate buffer—PBS (pH 7.4) containing EDTA to prevent cell aggregation. The collected samples were homogenized and subjected to total cell counting using a Neubauer chamber after appropriate dilution. In cases of high cell concentration, a 0.2% fuchsin solution was used to enhance visualization and facilitate cell distinction during counting.

### Histology Protocol

Subcutaneous tissue samples were fixed in 10% paraformaldehyde (PFA) in 0.1 M phosphate buffer—PBS (pH 7.4) for 24 hours, then dehydrated, bleached, and embedded in paraffin. Sections of 5 μm thickness were prepared and stained with hematoxylin and eosin (H&E) to assess inflammatory infiltrates. Thus, the tissue slides were examined under an optical microscope (Eclipse 50i, Nikon, Melville, NY, USA) at 200× magnification, with four fields captured per section. Images were acquired using a Nikon camera (Sight DS-5M-L1, Melville, NY, USA) and analyzed with NIH ImageJ 1.36b software (NIH, Bethesda, MD, USA) based on nuclear staining of inflammatory cells. Results were expressed as optical density (OD) [15].

### NFκB and IL-1β Gene Expression Analyses

Gene expression of NFκB and IL-1β was analyzed by real-time PCR (RT-PCR) as previously described by [15]. TRIzol® was used for total RNA extraction, and quantification was performed by spectrophotometry (Molecular Devices SpectraMax iD3 Multi-Mode Microplate Reader) at 260/280 nm, to ensure quality. cDNA synthesis was performed with *M-MLV* reverse transcriptase, and real-time polymerase chain reaction (PCR) amplification was monitored and quantified using SYBR Green dye system. The primer sequences used are described in Table 1.

**Table 1:** Primer sequences

GCGTCCACCCGCGAGTACAC	β-actin F
CGACGACGAGCGCAGCGATA	β-actin R
AGAAGCTTCCACCAATACTC	IL-1-β F
AGCACCTAGTTGTAAGGAAG	IL-1-β R
CGGGCATGCGTTTCCGTTA	NFκB F
TCTGTGCTTCTCTCCCCAGGAA	NFκB R

### Inflammatory Biomarkers

Cytokines TNF-α and IL-1β were quantified using a sandwich enzyme-linked immunosorbent assay (ELISA), according to the manufacturer’s instructions (Invitrogen ELISA Kit, Thermo Fisher Scientific—Bender MedSystems GmbH, Vienna, Austria). Homogenized subcutaneous tissue samples were processed and applied to a high-binding 96-well ELISA plate (Corning 3590), and absorbance at 450 nm was measured using a spectrophotometer.

### Oxidative Stress Analyses

#### Oxidants

ROS production was quantified by oxidation of 2’,7’-dichlorodihydrofluorescein diacetate (DCFH-DA) to 2’,7’-dichlorofluorescein (DCF). DCF levels were evaluated by samples incubated with H₂DCFDA (10 µM) for 30 min at 37°C in the dark, and the fluorescence was monitored at excitation and emission wavelengths of 488/525 nm, using a fluorescence spectrophotometer (Molecular Devices SpectraMax iD3 Multi-Mode Microplate Reader). The DCF standard curve (10 µM) was used as an internal control, and the results were expressed as relative fluorescence intensity [16]

Nitrite (NO₂⁻) levels were quantified by the Griess reaction, as described previously by Chae et al. [17]. The samples were incubated with the Griess reagent (1% sulfanilamide and 0.1% N-1 (naphthyl) ethylenediamine) at room temperature for 10 min, and the absorbance was measured at 540 nm. Nitrite concentration was calculated from a sodium nitrite standard curve (1–100 µM) and expressed in µM nitrite/mg protein.

#### Oxidative Damage Markers

Total thiol content was quantified using the DTNB (Ellman’s reagent or 5,5′-dithiobis-2-nitrobenzoic acid) method, as described by Aksenov and Markesbery [18]. Samples were incubated with 10 mM DTNB for 30 minutes at room temperature, and absorbance was measured at 412 nm. Thiol concentrations were calculated based on a standard curve (R^2^ ≥ 0.99) and expressed as µmol/mg of protein.

The carbonyl groups, through derivatization with 2,4-dinitrophenylhydrazine (DNPH), were used to assess protein oxidation, as carbonylated proteins, resulting in the formation of hydrazones. Proteins were first precipitated with 20% trichloroacetic acid (TCA), incubated with DNPH, and subsequently redissolved in 6 M guanidine. Absorbance was measured at 370 nm, and carbonyl content was expressed in nmol/mg of protein, using a molar extinction coefficient of 22,000 M⁻^1^·cm⁻^1^.

#### Antioxidant Enzyme Activities

SOD activity was determined by its ability to inhibit adrenaline auto-oxidation, following the method of Bannister and Calabrese [19]. Tissue samples were homogenized in glycine buffer, and aliquots of 5, 10, and 15 μL were incubated with catalase, glycine buffer, and adrenaline. The reaction proceeded for 180 seconds, and absorbance was read at 480 nm. Results were expressed as SOD units per milligram of protein (U/mg protein).

GSH levels were determined according to the method of Hissin and Hilf [20], with modifications. Samples were deproteinized with 10% TCA and incubated with phosphate buffer (pH 7.4) and 500 mM orthophthaldialdehyde. After 10 minutes, fluorescence was measured at 412 nm. GSH concentrations were calculated using a standard curve of reduced glutathione and expressed as fluorescence units per milligram of protein (U fluorescence/mg protein).

### Determination of Protein Content

Total protein content in homogenized tissue samples was measured by the Lowry et al. method [21]. Following adding the Folin–Ciocalteu reagent, absorbance was measured at 750 nm. Protein concentration was calculated using a bovine serum albumin standard curve and expressed as mg of protein.

## Results

### Subcutaneous Lavage Analysis

**Fig. 1:**
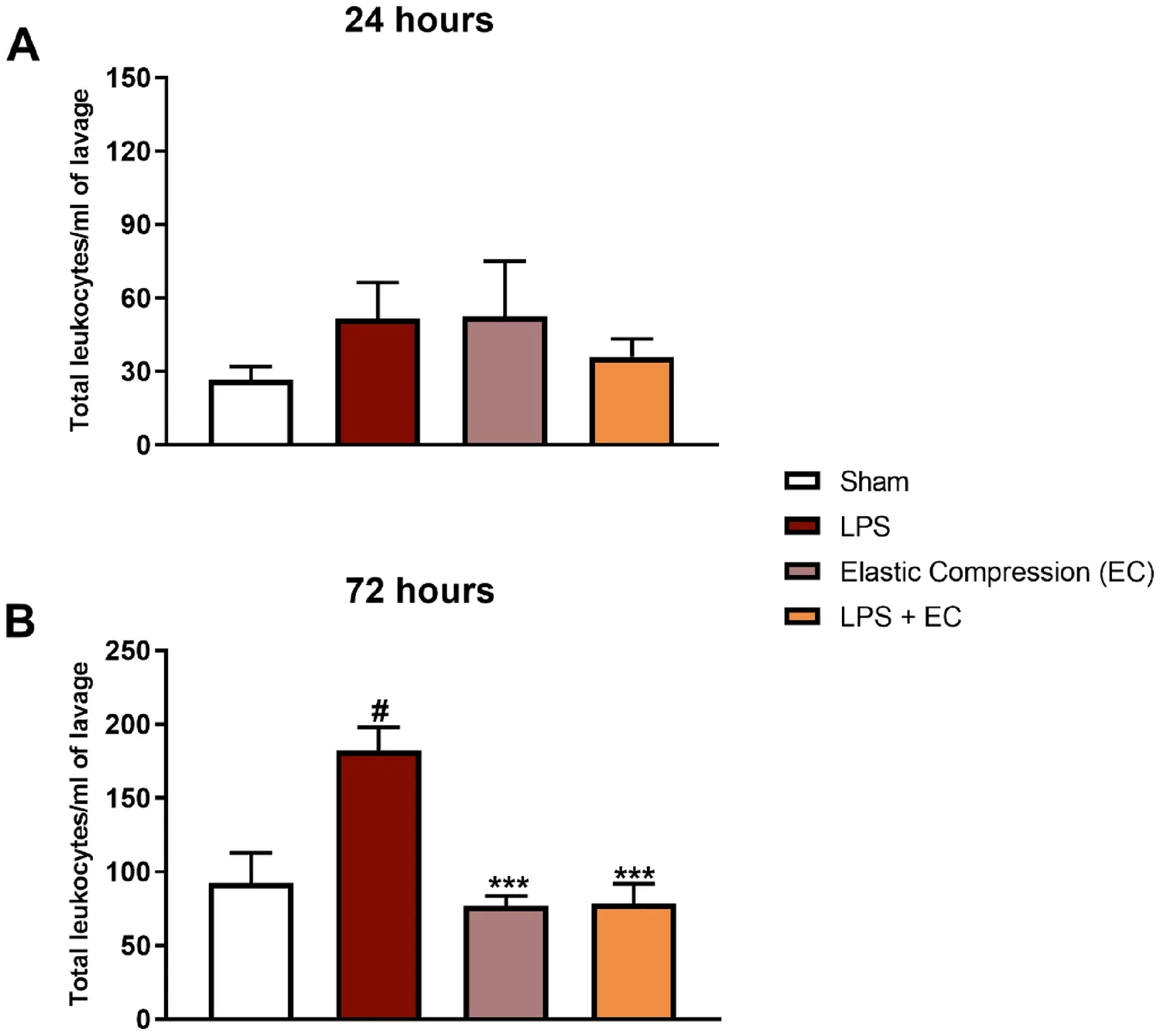
Effects of elastic compression treatment after LPS-induced inflammation on the total leukocyte count per mL/wash at 24 hours **A** and 72 hours **B**. Data are presented as mean ± SEM. *p* < 0.05 vs. LPS group; *p* < 0.01 vs. LPS group; *p* < 0.001 vs. LPS group; p < 0.0001 vs. LPS group; #*p* < 0.05 vs. Sham group (one-way ANOVA followed by Tukey’s post hoc test)

Subcutaneous exudate was collected via peritoneal lavage, and total leukocyte counts (cells/mL) were performed, as shown in Fig. 1. At the 24-hour time point, no significant differences were observed between experimental groups. However, at the 72-hour time point, the LPS group displayed a significant increase in leukocyte count compared to the Sham group (*p*<0.05). Meanwhile, the EC and LPS+CE groups showed a significant reduction in IL-1 expression compared to the LPS group (***p<0.001), as depicted in Fig. 1.

### Histological Analysis of the Inflammatory Infiltrate

**Fig. 2:**
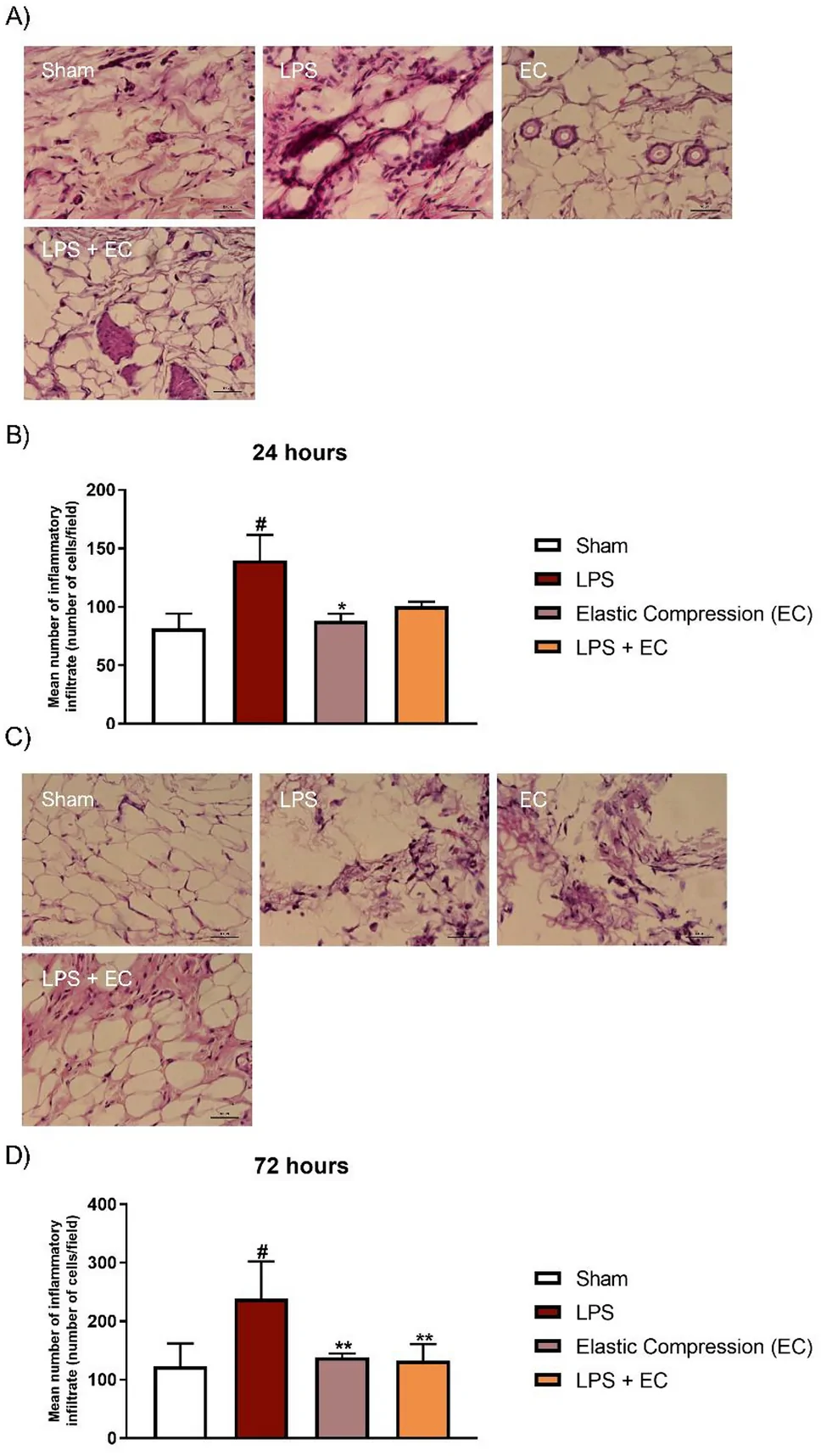
Effects of elastic compression treatment after LPS-induced inflammation on the histological analysis of subcutaneous tissue, with representative image at 24 hours **A** and corresponding statistical graph **B**, as well as representative image at 72 hours **C** and corresponding statistical graph **D**. Data are presented as mean ± SEM. *p* < 0.05 vs. LPS group; *p* < 0.01 vs. LPS group; *p* <  0.001 vs. LPS group; *p* < 0.0001 vs. LPS group; #*p* < 0.05 vs. Sham group (one-way ANOVA followed by Tukey’s post hoc test)

Histological examination revealed inflammatory cell infiltration in subcutaneous tissue at both 24 hours and 72 hours (Fig. 2). At 24 hours, the LPS group exhibited a significant increase in inflammatory infiltrate compared to the Sham group (*p*<0.05), while the EC group showed a significant reduction compared to LPS (*p*<0.05). The LPS+CE group demonstrated a profile similar to the Sham group (*p<0.01). At 72 hours, the LPS group maintained a significant increase in infiltration relative to Sham (*p*<0.05). In contrast, both EC and LPS+CE groups showed significantly reduced infiltrate compared to the LPS group, as illustrated in Fig. 2.

### NF-κB and IL-1β Gene Expression Analysis

**Fig. 3:**
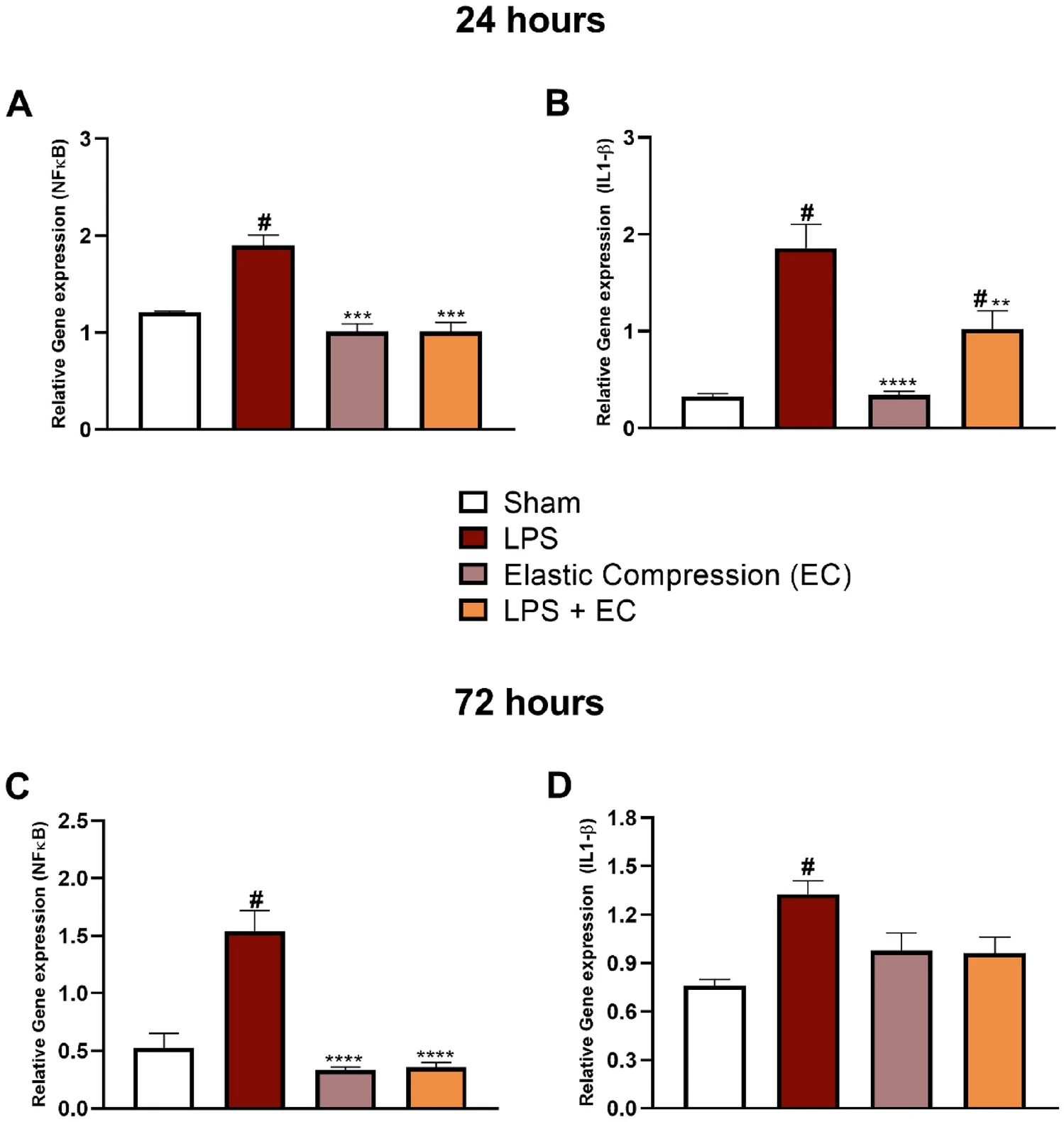
Effects of elastic compression treatment after LPS-induced inflammation on gene expression analysis of NF-κB at 24 hours **A** and 72 hours **C**, and IL-1β at 24 hours **B** and 72 hours **D**. Data are presented as mean ± SEM. *p* < 0.05 vs. LPS group; *p* < 0.01 vs. LPS group; *p* < 0.001 vs. LPS group; *p* < 0.0001 vs. LPS group; #*p* < 0.05 vs. Sham group (one-way ANOVA followed by Tukey’s post hoc test)

The evaluation of inflammatory biomarkers was performed using RT-PCR to analyze NF-κB and IL-1β gene expression. At 24 hours, NF-κB expression was significantly elevated in the LPS group compared to Sham group, while both EC and LPS+CE groups showed a significant reduction (***p<0.001) when compared to LPS, as shown in Fig. 3. This trend persisted at 72 hours, with EC and LPS+CE groups maintaining significantly lower expression levels (****p<0.0001) compared to the LPS group. For IL-1β at 24 hours, expression was significantly upregulated in the LPS group compared to Sham (*p*<0.05). The EC group showed a marked decrease in expression relative to LPS (****p<0.0001), and the LPS+CE group also exhibited reduced expression (*p*<0.01), with a significant difference from Sham (*p*<0.05), as illustrated in Fig. 3. At 72 hours, the LPS group continued to show increased expression compared to Sham group, while the other groups displayed no significant differences among themselves, resembling the Sham profile.

### Evaluation of Pro-Inflammatory Cytokines

**Fig. 4:**
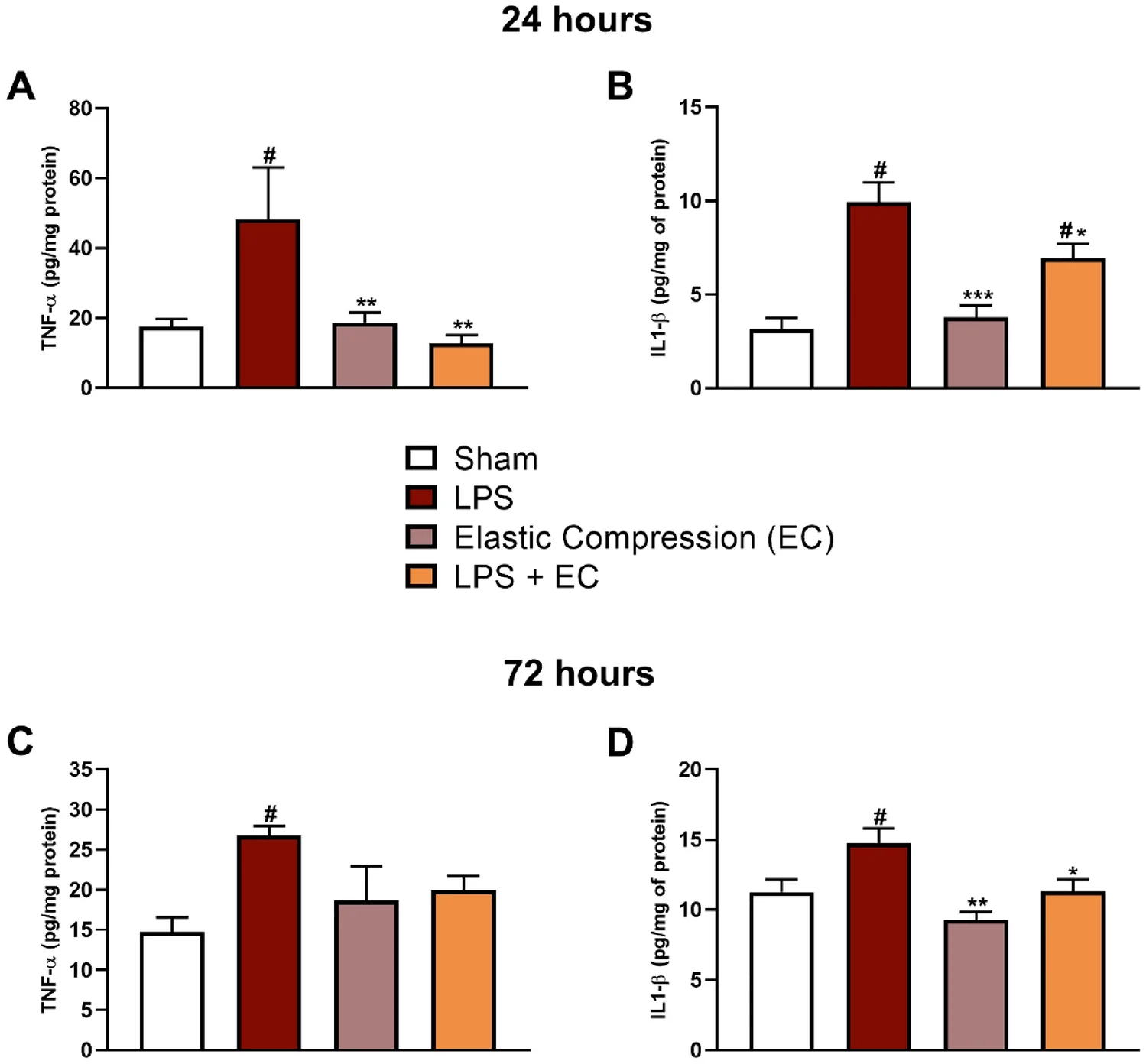
Effects of elastic compression treatment after LPS-induced inflammation on protein levels of TNF-α at 24 hours **A** and 72 hours **C**, and IL-1β at 24 hours **B** and 72 hours **D**. Data are presented as mean ± SEM. *p* < 0.05 vs. LPS group; *p* < 0.01 vs. LPS group; *p* < 0.001 vs. LPS group; *p* < 0.0001 vs. LPS group; #p < 0.05 vs. Sham group (one-way ANOVA followed by Tukey’s post hoc test)

As shown in Fig. 4, TNF-α protein levels were significantly increased in the LPS group at 24 hours compared to the Sham group, while EC and LPS+CE groups exhibited a significant reduction (*p*<0.01) compared to LPS. At 72 hours, only the LPS group maintained elevated TNF-α levels compared to the Sham group. The remaining groups showed no significant differences at this time point.

IL-1 levels at 24 hours (Fig. 4) were significantly elevated in the LPS group compared to the Sham group. The EC group exhibited a marked reduction in IL-1 levels compared to LPS (*p*<0.0001), and the LPS+CE group also showed a significant decrease compared to LPS (*p*<0.05), as well as a significant difference from Sham (*p*<0.05). At 72 hours, this reduction remained consistent and significant compared to the LPS group.

### Markers of Oxidative Stress

#### Oxidant, Damage, and Antioxidant Activity

**Fig. 5:**
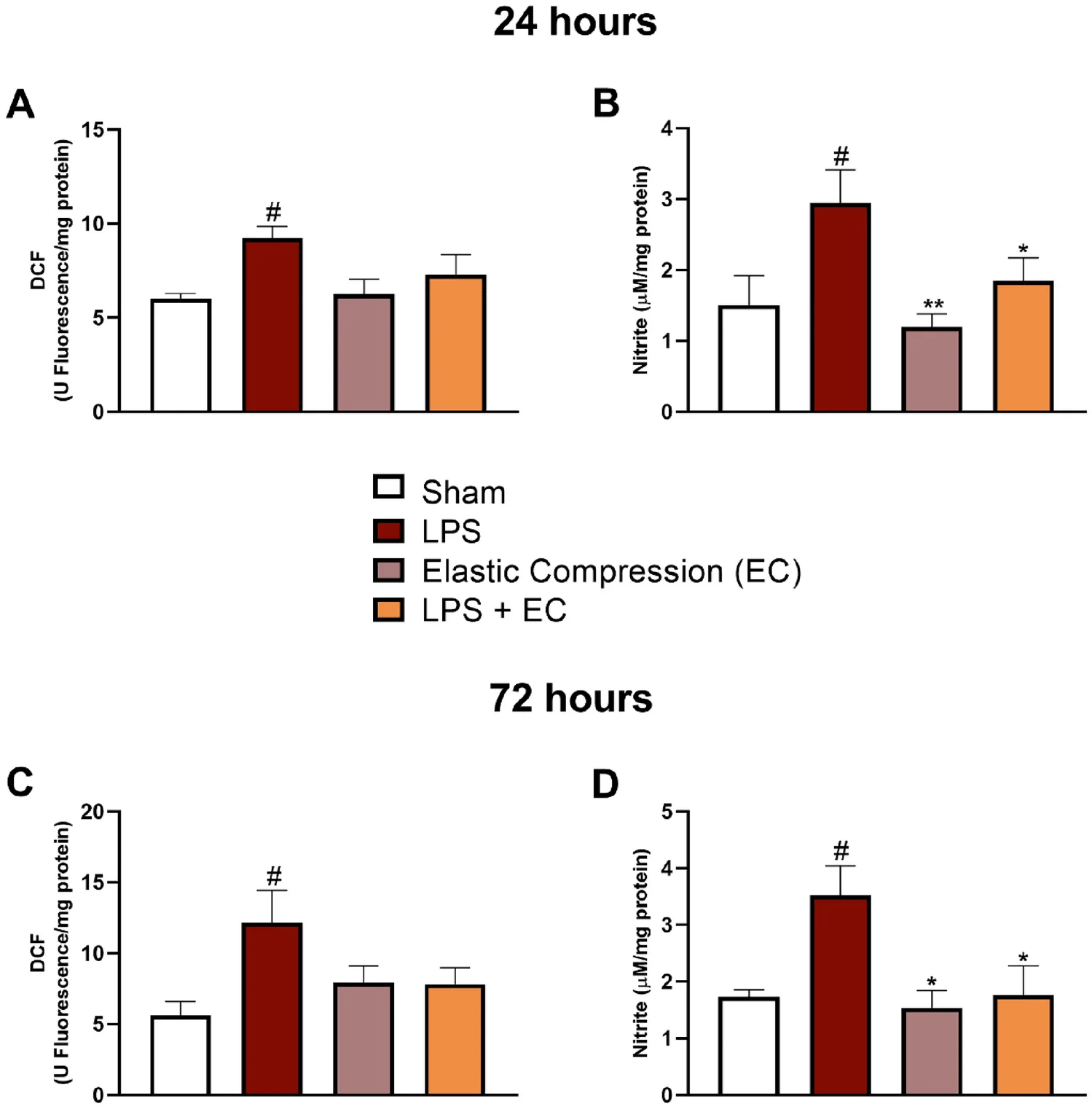
Effects of elastic compression treatment after LPS-induced inflammation on oxidant levels, with nitrite at 24 hours **B** and 72 hours **D**, and DCF levels at 24 hours **A** and 72 hours **C**. Data are presented as mean ± SEM. *p* < 0.05 vs. LPS group; *p* < 0.01 vs. LPS group; *p* < 0.001 vs. LPS group; *p* < 0.0001 vs. LPS group; #*p* < 0.05 vs. Sham group (one-way ANOVA followed by Tukey’s post hoc test)

Regarding nitrite levels, indicative of nitric oxide, at 24 hours, the LPS group showed a significant increase relative to the Sham group, whereas the EC and LPS+CE groups maintained levels comparable to the Sham, presenting no significant differences. At 72 hours, nitrite levels were again significantly elevated in the LPS group when compared to Sham, while EC (*p*<0.01) and LPS+CE (*p*<0.05) groups showed a significant reduction compared to LPS (see Fig. 5).

DCF levels (see Fig. 5), a marker of ROS, were significantly increased in the LPS group at 24 hours (*p*<0.05) compared to the Sham group, while the other groups showed no significant differences. At 72 hours, LPS maintained elevated DCF levels compared to Sham (*p*<0.05), while EC and LPS+CE groups demonstrated a significant reduction compared to LPS (*p*<0.05).

**Fig. 6:**
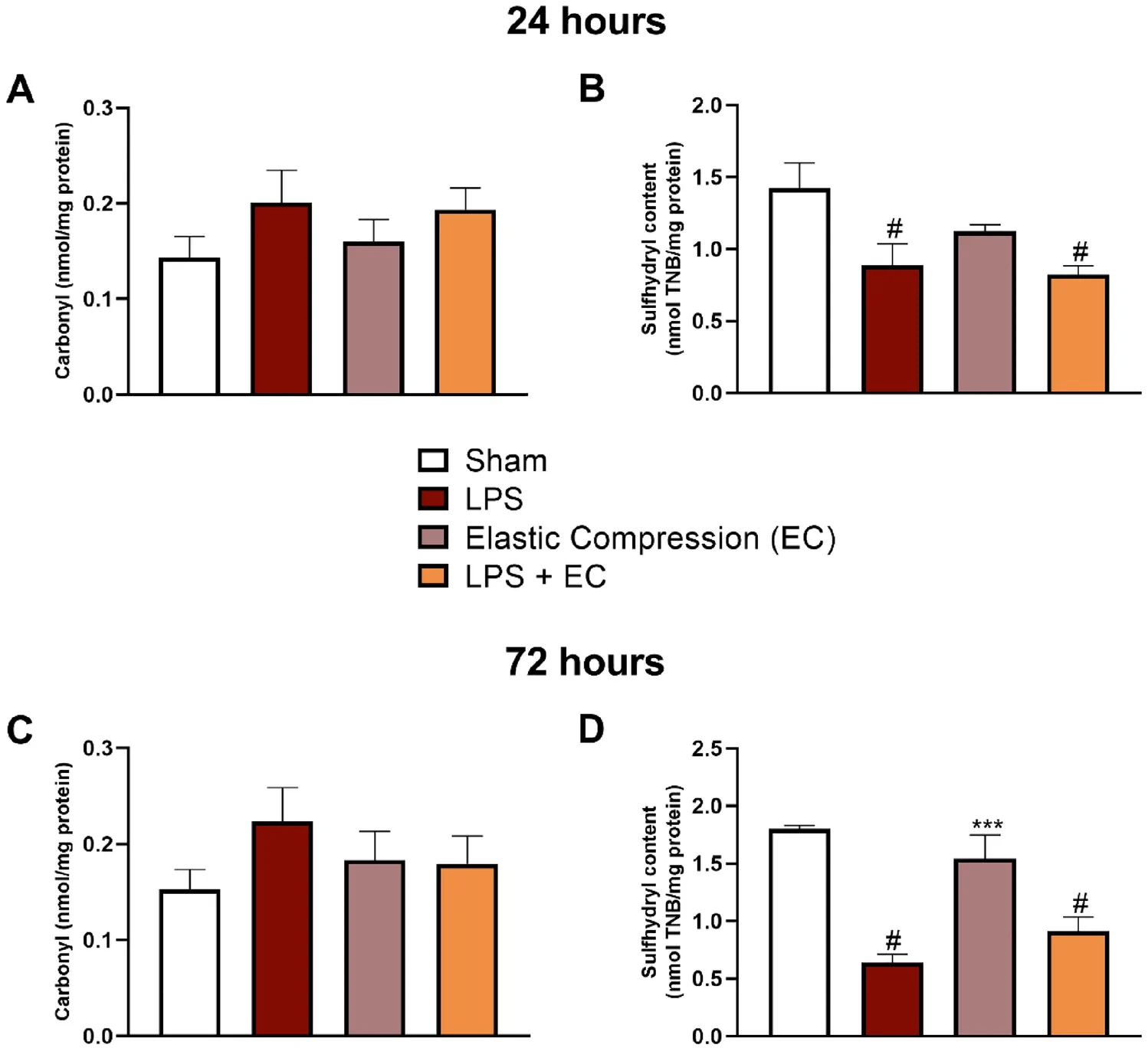
Effects of elastic compression treatment after LPS-induced inflammation on oxidative damage: protein carbonyl levels at 24 hours **A** and 72 hours **C**, and sulfhydryl content at 24 hours **B** and 72 hours **D**. Data are presented as mean ± SEM. *p* < 0.05 vs. LPS group; *p* < 0.01 vs. LPS group; *p* < 0.001 vs. LPS group; *p* < 0.0001 vs. LPS group; #*p* < 0.05 vs. Sham group (one-way ANOVA followed by Tukey’s post hoc test)

Assessment of oxidative damage through protein carbonylation (Fig. 6) revealed no significant differences between groups at either time point. Regarding thiol content (Figures 6), at 24 hours, both LPS and LPS+CE groups exhibited a significant decrease compared to Sham (*p*<0.05), whereas the EC group did not differ significantly. At 72 hours, LPS and LPS+CE groups continued to show reduced thiol levels compared to Sham, while the EC group presented a significant increase compared to LPS (*p*<0.001).

**Fig. 7:**
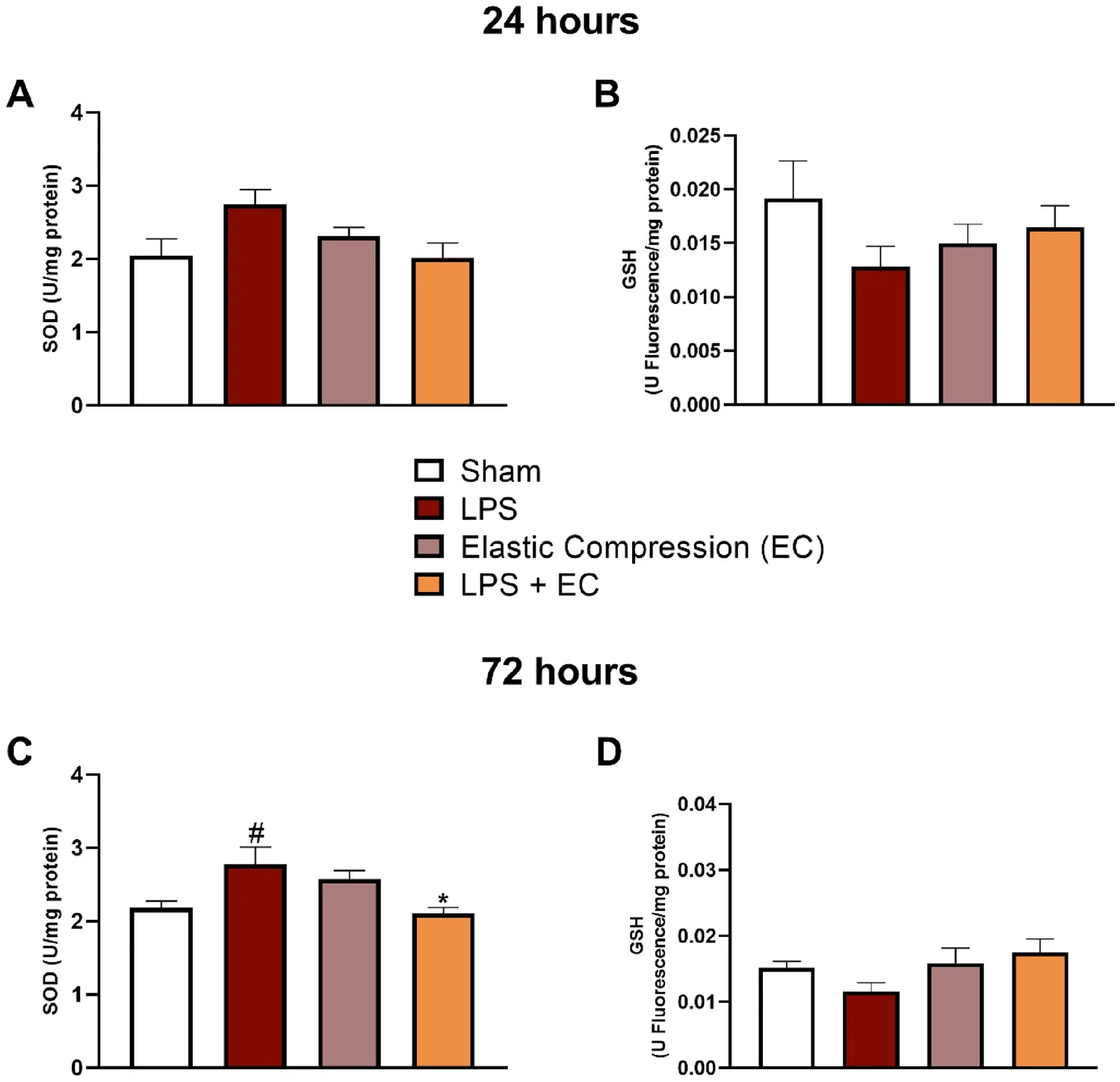
Effects of elastic compression treatment after LPS-induced inflammation on antioxidant defense: SOD levels at 24 hours **A** and 72 hours **C**, and GSH content at 24 hours **B** and 72 hours **D**. Data are presented as mean ± SEM. *p* < 0.05 vs. LPS group; *p* <  0.01 vs. LPS group; *p* < 0.001 vs. LPS group; *p* < 0.0001 vs. LPS group; #*p* < 0.05 vs. Sham group (one-way ANOVA followed by Tukey’s post hoc test)

In the antioxidant defense analysis, superoxide dismutase (SOD) activity showed no significant differences at 24 hours. However, at 72 hours, the LPS group demonstrated a significant increase in SOD activity compared to Sham (*p*<0.05), while the LPS+CE group showed a significant reduction relative to LPS (*p*<0.05), as illustrated in Fig. 7. Glutathione (GSH) levels did not differ significantly between groups at either time point (see Fig. 7).

## Discussion

In clinical contexts, acute inflammation is often accompanied by signs such as edema, erythema, and cellular infiltration, which, if not properly controlled, can progress to complications that negatively impact therapeutic outcomes [22, 23]. When exacerbated or prolonged, this response may compromise tissue integrity and delay functional recovery [24].

In this study, we employed a model of acute subcutaneous abdominal inflammation induced by lipopolysaccharide (LPS). Subcutaneous LPS administration triggered typical manifestations of the inflammatory process [25], as evidenced by a significant increase in leukocyte count in subcutaneous lavage at 72 hours, along with a marked influx of inflammatory cells observed in histological analyses at both 24 and 72 hours post-induction.

These effects are attributed to the ability of LPS to bind Toll-like receptors (TLRs), particularly TLR4, inducing a conformational change that triggers a complex intracellular signaling cascade [26, 27]. This process results in the activation of transcription factors such as nuclear factor kappa B (NF-κB) and activator protein-1 (AP-1), which regulate the expression of pro-inflammatory genes, cytokines, and chemokines [28]. Based on this model, we investigated the potential of elastic compression as a strategy to modulate acute inflammation.

Compression therapy is a non-pharmacological alternative widely applied in clinical practice, particularly for reducing postoperative edema and hematoma risk [9]; however, its role in controlling inflammation remains underexplored in the literature. Thus, in this study, we showed that elastic compression significantly reduced the total leukocyte count in subcutaneous lavage at 72 hours post-induction. Histological analyses also revealed a reduction in inflammatory cell infiltration at both evaluated time points, 24-hour and 72-hour.

The reduction in inflammatory infiltrate observed in this study is supported primarily by the compressive effect on edema, triggered by the vascular effects of the bandage [29]. Some studies suggest that this effect on exudate reduction is directly related to the compressive force exerted by the bandage, which secondarily leads to a decrease in inflammatory infiltrate [9, 29–32].

At the tissue level, this compressive effect is interpreted as a mechanical stimulus that induces hemodynamic changes, while also providing a physical barrier and mechanical support against mechanical stress, such as shear forces, thus preventing amplification of the inflammatory process [33]. According to Jokuszies et al. [34], edema reduction and consequent inflammatory control may also be related to activation of the veno-arterial reflex mechanism.

It is well known that the inflammatory infiltrate, particularly neutrophils and macrophages, releases factors that amplify the response and increase oxidative demand via phagocytosis. These cells also activate classical inflammatory pathways via Toll-like receptors and inflammasome signaling [35].

These inflammatory signals activate the canonical NF-κB signaling pathway, wherein IKK1/2 phosphorylates IκB proteins bound to NF-κB, targeting them for ubiquitination and proteasomal degradation [36, 37]. Free IκBs are also constitutively degraded via a ubiquitin-independent proteasomal pathway. As IκBs are degraded, NF-κB translocates to the nucleus, binds to κB sites in the DNA, and activates gene transcription [38, 39].

Consequently, many inflammatory cytokines (such as TNF and IL-1) are produced, which can further activate NF-κB via positive feedback loops. Moreover, the activated NLRP3 inflammasome processes pro-IL-1β and IL-18 into their biologically active mature forms, key cytokines that promote inflammation and coordinate both innate and adaptive immune responses [39, 40].

Mechanical forces exerted on cells can also activate NF-κB; shear stress (mechanotransduction) activates NF-κB via phospholipase pathways that increase intracellular Ca^2^⁺ levels. In turn, NF-κB activated by shear stress upregulates COX-2, a key player in response to mechanical load [38, 41]. In line with the observed reduction in inflammatory infiltrate, the LPS group treated with elastic bandage compression exhibited a significant reduction in NF-κB gene expression at both 24 and 72 hours, as well as a significant decrease in IL-1β gene expression at 24 hours.

To our knowledge, these findings are the first to demonstrate the ability of elastic compression to modulate inflammation at both microvascular and gene expression levels of inflammatory nuclear factors. Notably, the regulation of IL-1β gene expression is highly relevant, as Zhang and Hallock [5] report that inflammasome complexes are implicated in fibrogenesis of various organs, mainly through excessive IL-1β production. This cytokine can directly convert intrinsic organ cells into fibroblasts via cell surface receptors, resulting in collagen and ECM overproduction and ultimately fibrosis [42, 43].

Consistent with the observed reductions in NF-κB and IL-1β gene expression, we also found significantly decreased protein levels of TNF-α and IL-1β at 24 hours, an effect that persisted through 72 hours post-induction. According to Jang et al. [44] and Wang and He [45] production of IL-6, TNF-α, and IL-1β is characteristic of activated monocytes and M1 pro-inflammatory macrophages. Given the reduced infiltrate observed here, it is plausible that compression modulates macrophage phenotype.

This reduction in pro-inflammatory markers highlights the ability of the intervention to regulate tissue response to LPS. TNF-α, through its receptor I domain, associates with the TNFR1-associated death domain protein, which in turn activates NFκB and MAPK cascades, resulting in inflammation, tissue degeneration, host defense, and cell survival [44].

Similarly, extracellular IL-1β, upon binding to its receptor IL-1R, activates transcription factors NF-κB and p38 MAPK. IL-1 signaling begins with the formation of a complex between MyD88, IRAK, and IRAK-2, ultimately leading to expression of inflammatory genes, particularly those encoding IL-8, monocyte chemoattractant protein-1, and cyclooxygenase-2 [46, 47]. Furthermore, Mirza et al. [48] report that excess TNF-α and IL-1β levels are associated with persistent M1 macrophage infiltration, which impairs activation of healing-associated factors.

These factors emphasize the importance of disrupting the self-perpetuating pro-inflammatory cycle driven by TNF-α and IL-1β. Supporting our findings [49, 50] observed reduced inflammatory cytokines, including TNF-α and IL-1β, following compression therapy in cases of venous ulceration, along with elevated anti-inflammatory markers.

Some studies indicate that TLR4-associated signaling is mechanically regulated and that macrophages are sensitive to their physical environment [51]. This suggests that the mechanical properties of soft tissue, such as biological stimuli and elastic substrates, can influence macrophage polarization, thereby affecting pro-inflammatory mediator production [52].

This phenomenon is also supported by Fahy et al. [53], who demonstrated the ability of monocytes to respond to mechanical stimuli, showing that compressive mechanical load modulates macrophage polarization. Similar to primary human monocytes, compression increased IL-10 expression—a known anti-inflammatory cytokine that promotes differentiation of macrophages toward an anti-inflammatory phenotype [53].

Additionally, integrins are key structures that mediate mechanotransduction due to their cytoplasmic domain anchoring to the actin cytoskeleton and their extracellular domain interacting with ECM molecules [54]. Major mediators of biomechanical signal transduction include integrin–matrix interactions, G protein-coupled receptors, mechanosensitive ion channels, and cytoskeletal tension responses [55].

Once mechanical forces activate these transmembrane sensors, multiple interrelated signaling pathways are triggered, including calcium-dependent targets, nitric oxide signaling, mitogen-activated protein kinases (MAPKs), and phosphoinositide 3-kinase (PI3K) pathways [6]. PI3K/Akt activity, in turn, regulates cell proliferation, migration, growth, survival, and apoptosis. Once activated, this pathway enhances fibroblast motility and α-SMA expression, contributing to tissue regeneration [56].

A hallmark of inflammatory processes is oxidative stress, resulting from deregulated production of reactive oxygen species (ROS), primarily of mitochondrial origin or via NADPH oxidase activation. In this study, elastic compression maintained basal levels of oxidative markers and nitrite at 24 hours post-induction; however, at 72 hours, these markers were significantly reduced compared to the LPS group. Regarding oxidative damage, only thiol content indicated loss of homeostasis at both time points. For antioxidant response, only the LPS group treated with an elastic bandage showed a significant reduction in SOD activity at 72 hours post-induction.

Some authors report that mechanotransduction influences cellular redox balance through mitochondrial dynamics mechanisms. As mitochondria are anchored to cytoskeletal structures, it is plausible that externally applied forces may be directly transmitted to mechanosensors on the mitochondrial surface [57].

Given that persistent acute inflammation—with infiltration of polymorphonuclear cells such as neutrophils and phagocytic macrophages, pro-inflammatory mediators, and an oxidative environment—is associated with postoperative complications, delayed wound healing, pain, and especially fibrosis, the effects observed in this study support the cellular and molecular efficacy of an already clinically applicable intervention. Our findings highlight the consistency of elastic compression materials in modulating inflammatory infiltrate, inflammatory gene and protein expression, and redox homeostasis.

Although an anti-inflammatory effect was observed in the study outcomes, several limitations should be acknowledged. First, the experimental model relies on a single acute inflammatory stimulus and therefore fails to fully recapitulate the temporal and biological complexity of inflammation induced by surgical trauma and its associated complications, such as edema/seroma formation and fibrosis. Additionally, although elastic compression was standardized through the application of Theraband Kinesiology Tape® with 50% stretch, the actual pressure exerted on the tissue (in mmHg) was not directly quantified. While our findings support an association between elastic compression and attenuation of inflammatory markers, the underlying signaling pathways and mechanistic bases—particularly those related to microvascular/hemodynamic effects and mechanotransduction signaling—remain to be elucidated. Future studies are warranted to clarify these mechanisms and to strengthen the translational foundation of this intervention in clinical settings.

## Conclusion

**Fig. 8:**
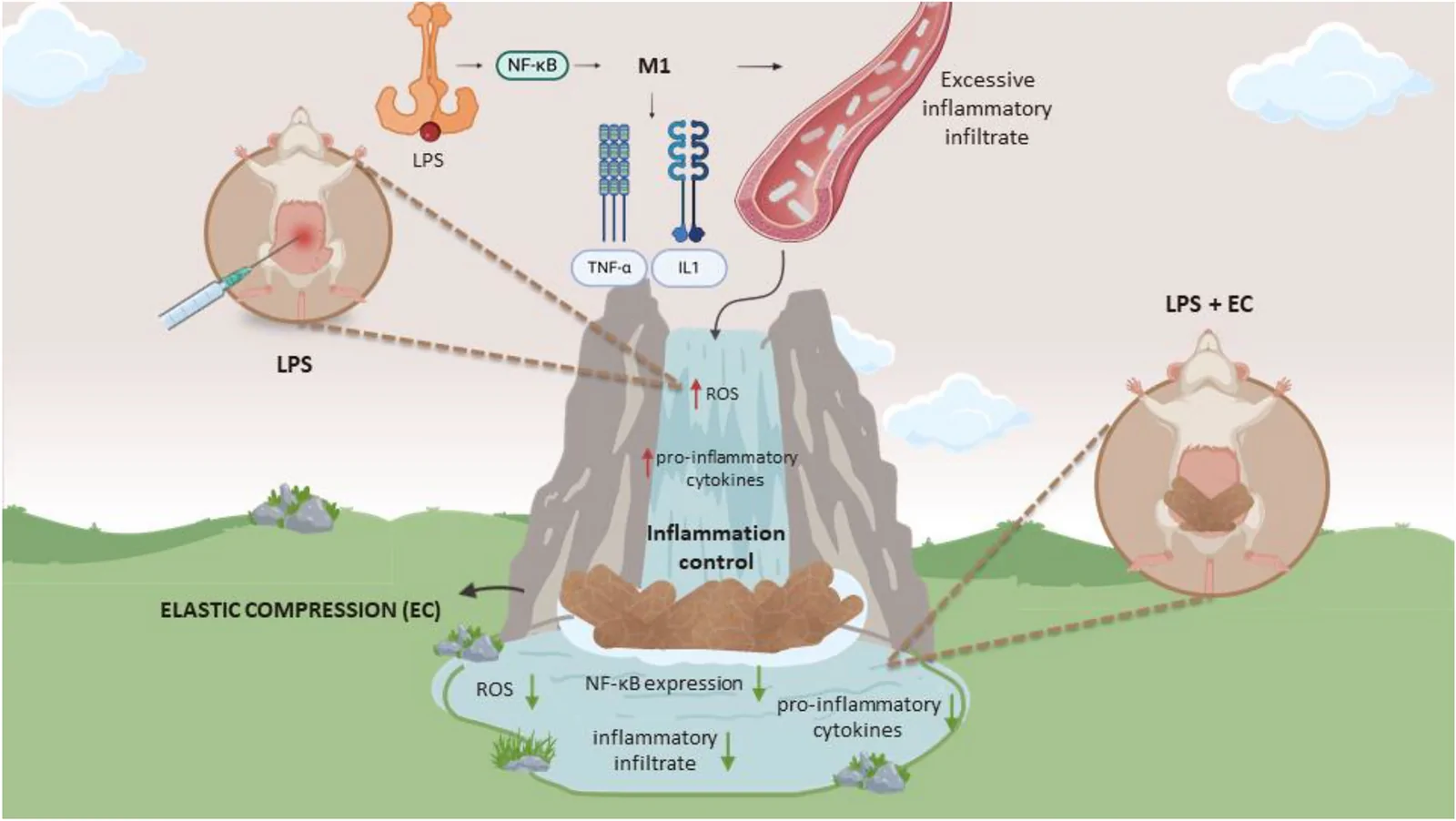
Schematic representation of the mechanism of action of elastic compression in modulating the inflammatory response. Inflammation without elastic compression (left) is initiated by the activation of M1 macrophages via LPS and NF-κB, leading to an excessive inflammatory infiltrate, edema, pain, and loss of function. This process is characterized by increased production of pro-inflammatory cytokines (TNF-α, IL-1) and reactive oxygen species (ROS). In contrast, elastic compression (right) acts as a mechanical barrier, regulating the inflammatory response. This results in reduced NF-κB expression, decreased production of pro-inflammatory cytokines and ROS, and consequently, a reduction in the inflammatory infiltrate. This mechanism suggests that elastic compression may mitigate the adverse effects of inflammation. Source: The author, 2025

The findings of this study demonstrate that elastic compression, when applied in an animal model of acute subcutaneous inflammation induced by LPS, can significantly modulate the inflammatory response, ranging from the reduction of cellular infiltration and edema to decreased gene and protein expression of pro-inflammatory markers, such as NFκB, TNF-α, and IL-1β. Mechanical intervention through compression proves effective in interrupting the inflammatory cycle, reinforcing the potential of elastic compression as a non-pharmacological therapeutic strategy for managing acute inflammation. This is a pioneering study that demonstrates the effects of elastic compression and paves the way for its broader applicability. Its potential use may extend to clinical contexts such as postoperative care, traumatic edema, and other inflammatory conditions affecting soft tissues. However, further translational and clinical research is needed to deepen our understanding of the underlying mechanisms, confirm its efficacy in humans, and establish evidence-based protocols for its application (Fig. 8).

## Data Availability

The data that support the findings of this study are available from the corresponding author, [Silveira, PCL], upon reasonable request.

## Declarations

### AI Use Declaration

In accordance with the ethical publication standards outlined by the Committee on Publication Ethics (COPE), we hereby declare that no generative artificial intelligence tools were used at any stage of the development of this manuscript, including writing, data analysis, interpretation, or image creation. All intellectual contributions are solely the work of the listed authors. The authors take full responsibility for the content and originality of this submission.

### Evidence-Based Medicine

As this is an experimental study conducted in an animal model, the classification by level of evidence is not applicable. This type of categorization is primarily intended for clinical studies involving human participants. Therefore, basic science, experimental research, animal studies, and cadaveric models do not require the assignment of an evidence level, in accordance with the principles of Evidence-Based Medicine.
